# Soybean plants expressing the *Bacillus thuringiensis cry8-*like gene show resistance to *Holotrichia parallela*

**DOI:** 10.1186/s12896-019-0563-1

**Published:** 2019-10-15

**Authors:** Di Qin, Xiao-Yi Liu, Cristina Miceli, Qi Zhang, Pi-wu Wang

**Affiliations:** 1 Biotechnology Center of Jilin Agricultural University, Jilin Agricultural University, Changchun, 130118 People’s Republic of China; 2 School of Biosciences and Veterinary Medicine, University of Camerino, MC, Camerino, Italy; 3 College of Life Sciences, Kyung Hee University, 1732, Deogyeong daero, Giheung-gu, Yongin-si, Gyeonggi-do 17104 Republic of Korea

**Keywords:** *Holotrichia parallela*, *Cry8-*like, *Bacillus thuringiensis* endotoxin, Transgenic soybean

## Abstract

**Background:**

*Cry8-*like from *Bacillus thuringiensis* (*Bt*) encodes an insecticidal crystal (Cry) protein. *Holotrichia parallela* (Coleoptera: Scarabaeoidae), commonly known as the dark black chafer, is a troublesome pest of soybean (*Glycine max*). To test whether *cry8-*like can confer resistance against *H. parallela* to soybean, we introduced *cry8-*like from the *Bt* strain HBF-18 into soybean cultivar Jinong 28.

**Results:**

Quantitative reverse transcription-PCR analysis demonstrated that *cry8-*like was expressed most highly in soybean leaves. In addition, Southern blot assays revealed that one copy of the integrated fragment was present in the transformed plants. Eight independent *cry8-*like transgenic lines were subsequently fed on by *H. parallela*. Under *H. parallela* feeding stress, the survival rates of the non-transgenic plants were 92% lower than those of the transgenic plants. The mortality rate of *H. parallela* increased when the larvae fed on the roots of T_1_ transgenic soybean plants. Moreover, the surviving larvae were deformed, and their growth was inhibited.

**Conclusions:**

Collectively, our data suggest that transgenic soybean plants expressing the *cry8-*like gene are more resistant to *H. parallela* than non-transgenic plants and that transgenic expression of the *cry8-*like gene may represent a promising strategy for engineering pest tolerance. The events generated in this study could thus be utilized in soybean breeding programs.

## Background

Soybean (*Glycine max* L. Merr.) is a globally important crop species that is grown for its oil and protein; moreover, its seeds are used for human consumption, animal feed, and industrial raw materials [1]. Soybean is self-pollinated, and soybean seeds are rich in protein (38–40%), in addition, soybean crops are utilized in crop rotations because of the ability of the plants to increase the amount of nitrate in the soil via symbiotic nitrogen fixation. However, soybean yields are severely affected by insects [2].

**Fig. 1:**
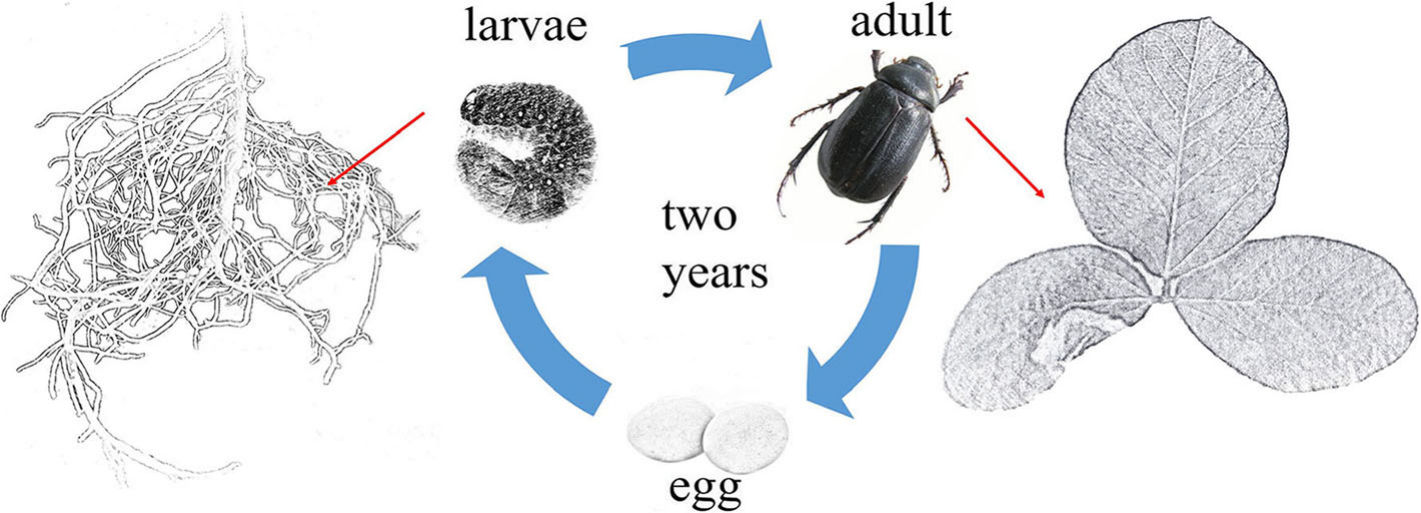
Three-stage life cycle of *Holotrichia parallela*

*Holotrichia parallela* (Coleoptera: Scarabaeoidea), commonly known as the dark black chafer, is one of the most important pests in agriculture and forestry in China [3]. *H. parallela* has a three-stage life cycle: the egg, larva and adult (Fig. 1). The larvae of *H. parallela* live in the soil and prefer to feed on plant roots during their two-year larval stage, and adult *H. parallela* insects feed on fresh leaves. *H. parallela* can cause considerable damage to soybean—from 10 to 30% yield losses in a typical year and up to 50% yield losses during severe infestations—and are responsible for $1–2 billion in losses per year worldwide [4]. Currently, farmers apply insecticidal sprays for the control of these insect pests, however, because these larvae are soil dwelling, it is difficult to find a specific chemical product that can control these insects.

Conventional breeding approaches for insect resistance have limited success due to a lack of sources of resistance. As an alternative approach, genetic engineering using *cry* genes from the soil bacterium *Bacillus thuringiensis* (*Bt*) offers great potential for improving crop species [5, 6].

*Bt* genes encode a protoxin that is transformed to an active toxin via enzyme cleavage in the insect midgut [7]. Different types of *cry* genes have been used successfully to develop insect-resistant crop species. According to insecticidal specificity, *cry* genes are mainly divided into 4 types including anti-Lepidoptera, anti-Diptera, anti-Coleoptera and anti-nematodes types. The *Bt* genes that provide resistance to Coleoptera include *cry3*, *cry4*, *cry7*, *cry8*, and *cry23* [8]. Development of insect resistance via transgenesis with fused *cry* genes has been previously applied to protect cotton from damage to cotton bollworm insects [9]. A novel *cry8* gene exhibiting activity against the larvae of *Holotrichia oblita* and *H. parallela* was identified [10]. Furthermore, a chimeric *cry8Ea1* gene flanked by MARs in transgenic peanut plants effectively controls *H. parallela* [11], but no studies have investigated transgenic soybean plants expressing *cry8*-like genes.

Therefore, in the present study, we used *Agrobacterium*-mediated transformation to transform callus of the soybean cultivar Jinong 28. Jinong 28 has good agronomic characteristics but no resistance to *H. parallela*. We produced eight independent transgenic soybean lines expressing *cry8-*like under the control of the CaMV 35S promoter. Insect feeding assays performed for a period of 96 h indicated effective protection of the soybean plants against *H. parallela* compared to the Jinong 28 control plants.

## Results

### Production of transgenic soybean plants expressing the cry8-like gene

**Fig. 2:**
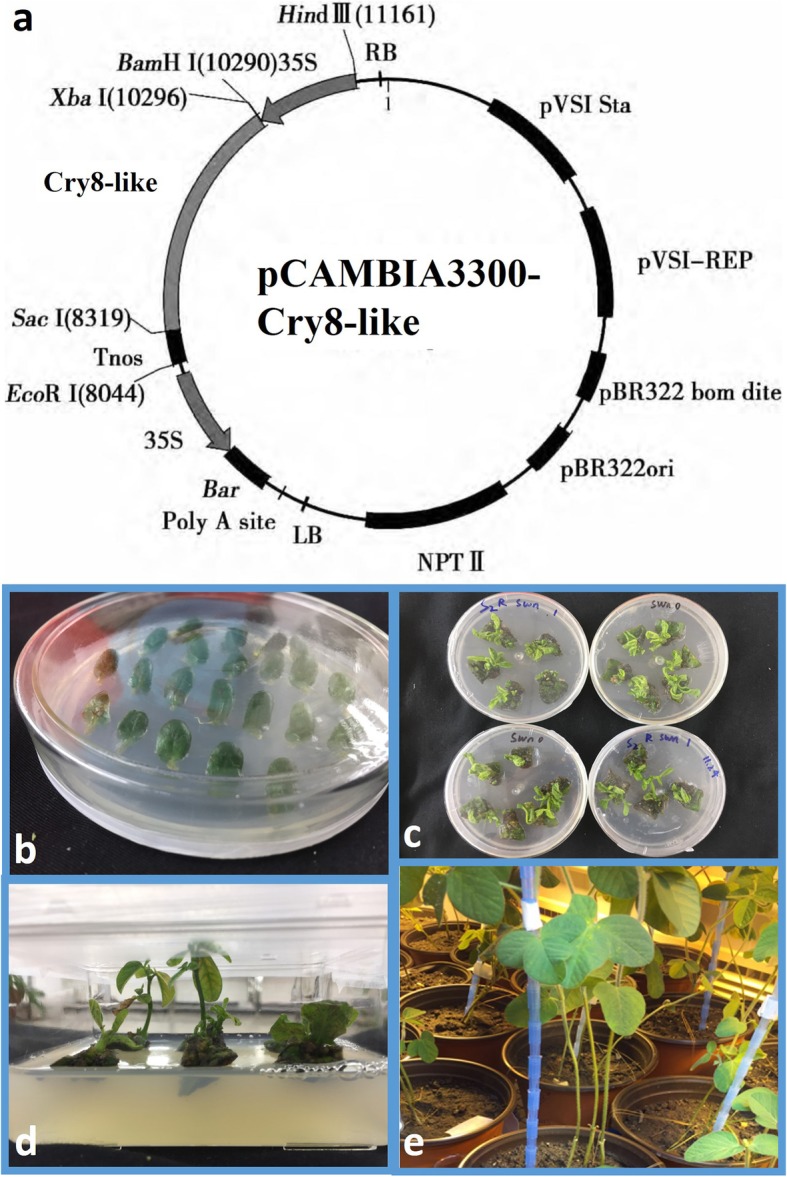
*Agrobacterium*-mediated transformation of soybean. (**a**) Diagram of pCAMBIA3300-cry8-like used for transformation. (**b**) Soybean cotyledon nodes cultured on shoot-induction medium (**c**) and (**d**) Shoot development. (**e**) 3-month-old T_0_ transgenic soybean plants grown under greenhouse conditions in soil

The recombinant plasmid designated pCAMBIA3300-cry8-like was introduced into *Agrobacterium tumefaciens* strain (Fig. 2a). The callus tissue used was originally derived from cotyledon-nodes of soybean (Fig. 2b). About 300 soybean cotyledon calluses were subjected to transformation. The putative transgenic embryos were grown with the addition.

of 50 mg·L^− 1^ kanamycin to select the transgenic plants. Approximately 3% of the stems (8 plants) developed on the kanamycin-selected cotyledons (Fig. 2c, d). 8 individual T_0_ plants (primary transformants) established in the green house that grew normally, flowered and set seeds (Fig. 2e). Since the transformants developed by the transformation strategy used could be chimeras in the T_0_ generation, screening of T_1_ generation plants for the identification of putative transformants was essential.

### Detection of the cry8-like gene in the T_0_/T_1_ generations

**Fig. 3:**
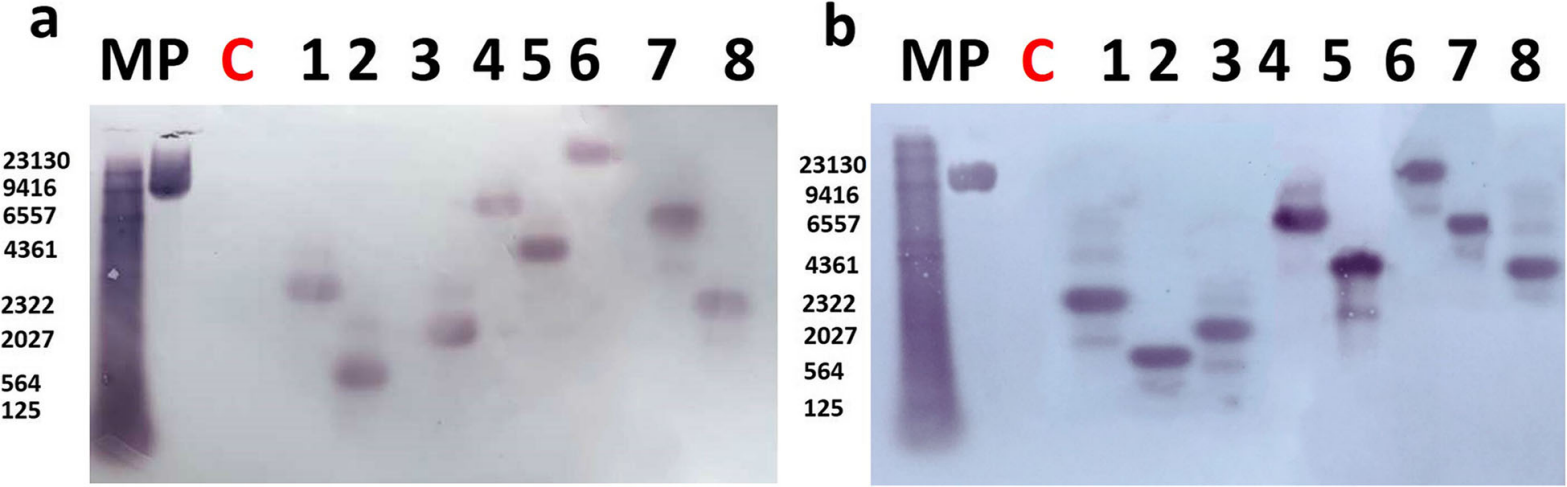
Presence of the *cry8*-like gene in different generations. (**a**) Southern blot analysis of the copy number of the *cry8*-like expression cassette in T_0_ plants. (**b**) Copy number of the *cry8*-like expression cassette in T_1_ plants. M: DNA marker; c: Jinong 28 controls

Southern blot assay was used to detect the presence and determine the copy number of the *cry8*-like gene in the selected eight putative transgenic lines. One copy of *cry8*-like was detected in T_0_ generation of each line (Fig. 3a). T_1_ generation of transgenic lines were obtained by selfing and inheritance of *cry8*-like investigated by PCR and Southern blot assay. PCR analysis of 971 plants revealed a 3:1 segregation ratio indicating that the transgene was stably integrated in the soybean genome. Plants of the T_1_ generation retained the single copy number of *cry8*-like insertion (Fig. 3b) and both in the T_0_ and T_1_ generations, each part of the transgene (*cry8*-like coding region, 35S promoter and *Nos* terminator) was detected in each selected line (Additional file 1: Fig. S1).

### Detection of the cry8-like expression in the transgenic lines

**Fig. 4:**
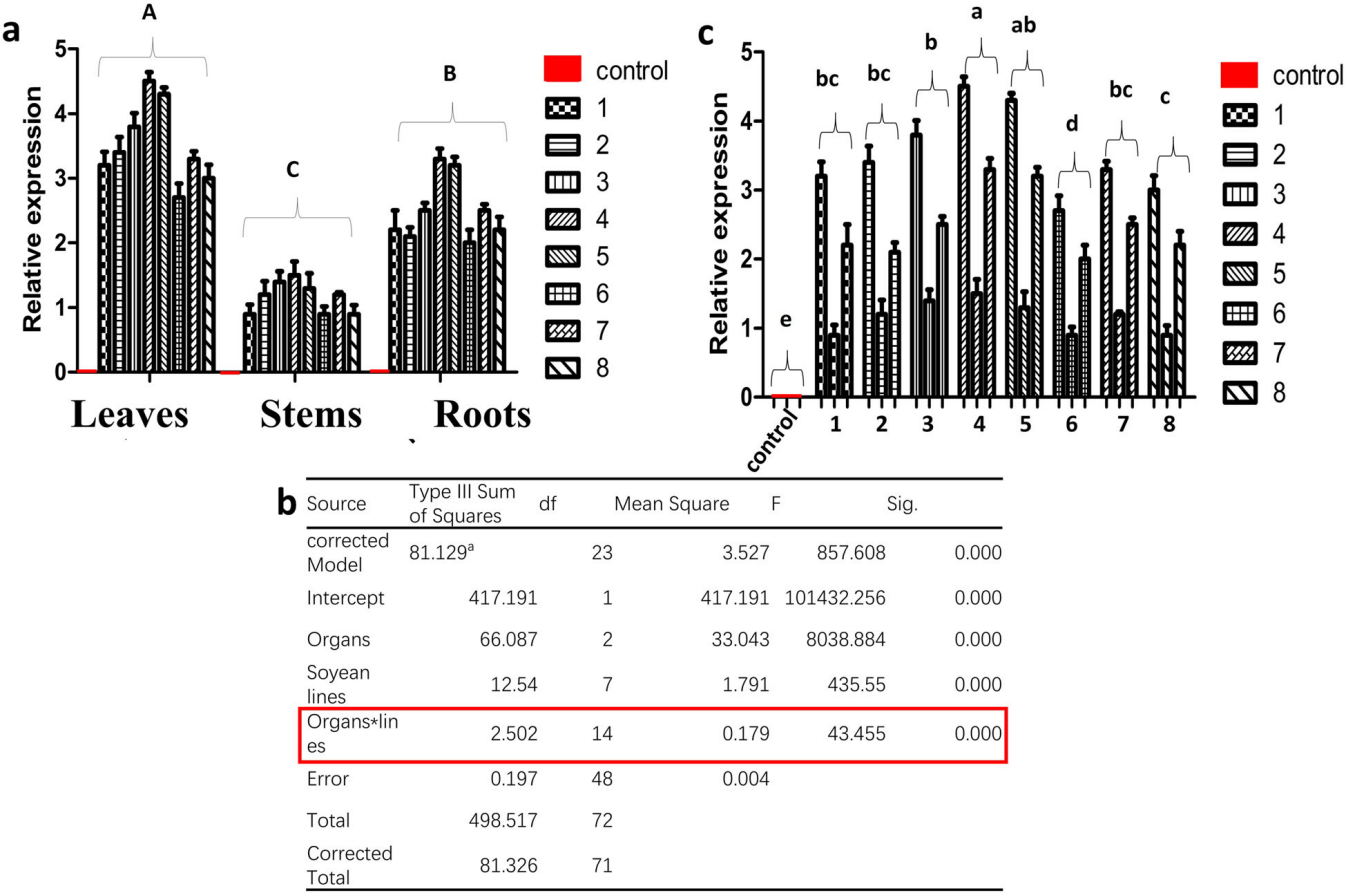
Detection of *cry8*-like expression by quantitative real-time PCR (qRT-PCR). (**a**) *cry8*-like expression in different organs of the eight transgenic lines (1–8) and the non-transformed control (red). (**b**) Test of between-subject effects. F: F-value; Sig: significant. (**c**) Comparison of the *cry8*-like expression levels of the eight transgenic lines. Different letters indicate significant differences at *P* < 0.05

Expression of *cry8*-like was studied by quantitative real-time PCR (qRT-PCR). The qRT-PCR results indicated that *cry8*-like was expressed in all organs (leaves, stems, and roots) of the transgenic soybean plants, with the highest expression level detected in the leaves (Fig. 4a). Two-way ANOVA (including all interactions and simple and main effects) was used to test for significant differences among the different organs and lines. The interaction between soybean lines and organs had a significant effect on the expression of *cry8*-like (Fig. 4b red), but the F-value of the interaction was small compared to that of the main effects. The results indicated that the simple and main effects of the lines and organs on *cry8-*like expression were more significant than the interaction effects. Two-way ANOVA revealed that there was a difference among the different *cry8*-like transgenic lines (*df* = 7, *F* = 435, *P* < 0.001) and the different organs (*df* = 2, *F* = 8033, *P* < 0.001) (Fig. 4b). The expression level of *cry8*-like in the eight lines followed the order Jinong 28-*cry*-4>Jinong 28-*cry*-5>Jinong 28-*cry*-3>Jinong 28-*cry*-2 ≥ Jinong 28-*cry*-1 ≥ Jinong 28-*cry*-7 ≥ Jinong 28-*cry*-6>Jinong 28-*cry*-8 (Fig. 4c).

### Quantifying the level of cry endotoxin in the different organs

An enzyme-linked immunosorbent assay (ELISA) was used to quantify the level of Cry endotoxin in the different organs (leaves, stems, and roots) of the transgenic soybean plants (T_0_/T_1_ generation). Variable amounts of toxin accumulated in all transgenic plants and in all organs, whereas the nontransformed control plants did not show any accumulation of Cry endotoxin (Table 1). The Jinong 28-cry-4 transgenic plants showed the highest level of Cry protein accumulation (see Additional file 2: Table S1). The ELISA also revealed that high levels of endotoxin accumulated in the leaves and roots and that the lowest level of endotoxin accumulated in the stems (see Additional files 3: Table S2). The interaction between soybean lines and organs had a significant effect on the accumulation of BT protein (Table 2). Compared with that of the main effects, the F-value of the interaction is small, and the results indicate that the simple effects of the lines and organs on Bt toxin accumulation were more significant than were the main effects.

**Table 1:** Bt toxin accumulation in the transgenic plants

Plant line (T_0_)	Bt toxin expression (ng·mg^−1^) in leaves	Bt toxin expression (ng·mg^− 1^) in stems	Bt toxin expression (ng·mg^− 1^) in roots	Plant line (T_1_)	Bt toxin expression (ng·mg^− 1^) in leaves	Bt toxin expression (ng·mg^− 1^) in stems	Bt toxin expression (ng·mg^− 1^) in roots
T_0_/Jinong28	–	–	–	T_1_/Jinong28	–	–	–
T_0_/Jinong28-cry-1	12 ± 1	9 ± 1	10 ± 2	T_1_/Jinong28-cry-1	13 ± 2	11 ± 1	12 ± 1
T_0_/Jinong28-cry-2	15 ± 2	11 ± 2	14 ± 2	T_1_/Jinong28-cry-2	11 ± 1	8 ± 1	10 ± 2
T_0_/Jinong28-cry-3	14 ± 2	11 ± 1	13 ± 1	T_1_/Jinong28-cry-3	18 ± 1	15 ± 1	16 ± 2
T_0_/Jinong28-cry-4	17 ± 2	12 ± 1	16 ± 2	T_1_/Jinong28-cry-4	19 ± 1	17 ± 2	18 ± 1
T_0_/Jinong28-cry-5	15 ± 2	10 ± 2	14 ± 1	T_1_/Jinong28-cry-5	17 ± 2	15 ± 2	17 ± 1
T_0_/Jinong28-cry-6	8 ± 2	5 ± 2	6 ± 1	T_1_/Jinong28-cry-6	10 ± 2	6 ± 1	8 ± 2
T_0_/Jinong28-cry-7	11 ± 1	7 ± 2	9 ± 3	T_1_/Jinong28-cry-7	12 ± 2	9 ± 2	10 ± 2
T_0_/Jinong28-cry-8	6 ± 2	5 ± 1	5 ± 1	T_1_/Jinong28-cry-8	8 ± 1	5 ± 2	6 ± 1

Note: Expression of Bt toxin in T_0_/T_1_ transgenic plants by ELISA in leaves, stems and roots. Results are means ± SE

**Table 2:** Test results of between-subject effects

Source	Type III Sum of Squares	df	Mean Square	F	Sig.
corrected Model	1355.259a	26	52.125	16.855	0.000
Intercept	5622.241	1	5622.341	1817.97	0.000
Organs	66.704	2	33.252	10.784	0.000
lines	1273.295	8	159.241	51.491	0.000
Organs*lines	14.672	16	1.194	1.282	0.003
Error	83.576	27	3.093		
Total	7061	54			
Corrected Total	1438.759	53			

Note: F: F value, Sig: significance

### Tolerance of the cry8-like transgenic plants to *H. parallela*

**Fig. 5:**
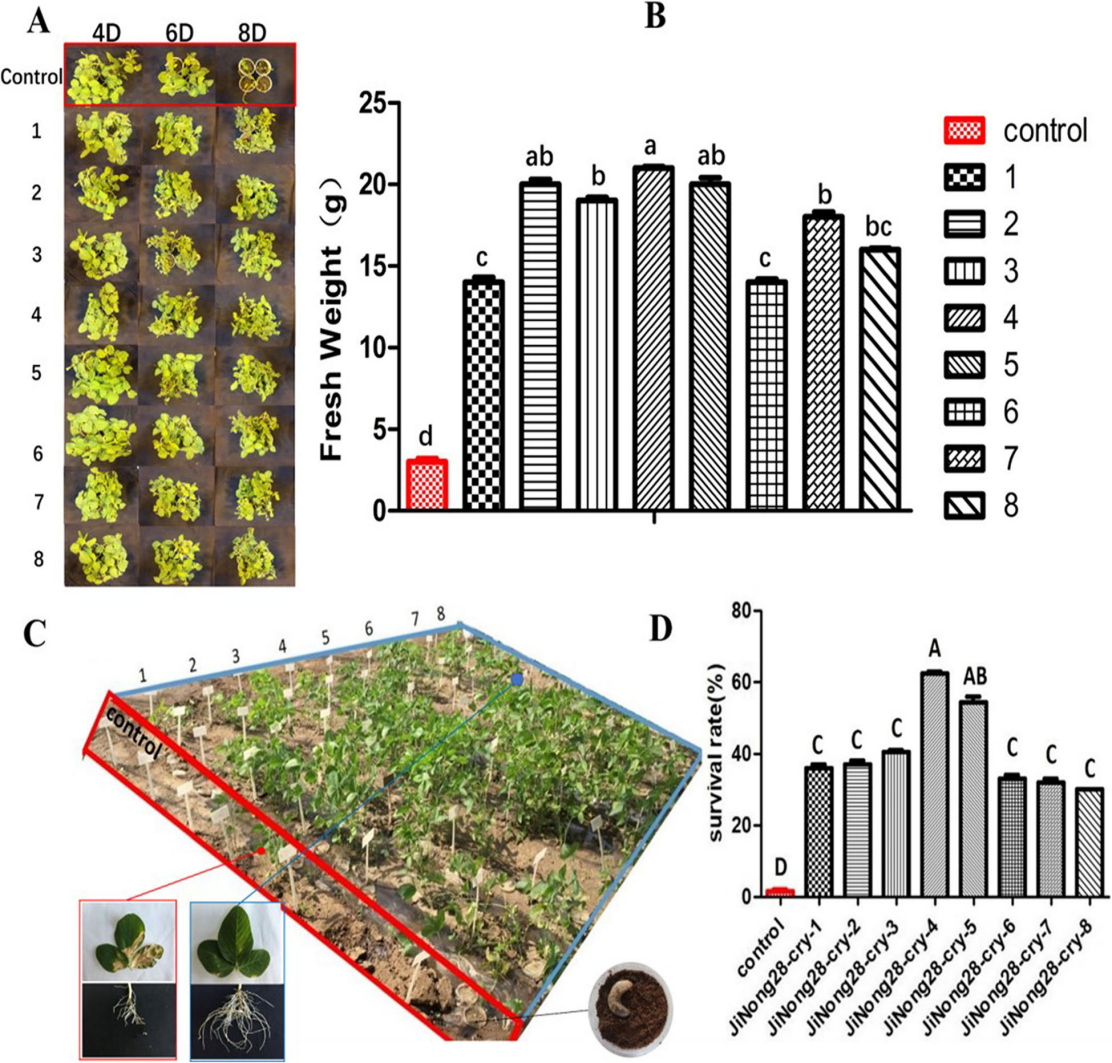
Tolerance of the *cry8*-like transgenic plants to *H. parallela*. (**a**) Representative images of the insect bioassay performed on transgenic soybean plants with *H. parallela* larvae after 4, 6, and 8 days of feeding. (**b**) Fresh weight of soybean plants after 8 days of *H. parallela* larval feeding. The bars represent the means ± standard errors; the mean values followed by the different letters are significantly different (*P* < 0.05), as determined by Duncan’s multiple-range test. (**c**) Phenotypes of 25-day-old plants treated with *H. parallela* for 14 days. Control: Jinong 28; 1–8: Jinong 28-cry-1, Jinong 28-cry-2, Jinong 28-cry-3, Jinong 28-cry-4, Jinong 28-cry-5, Jinong 28-cry-6, Jinong 28-cry-7, and Jinong 28-cry-8; (**d**) Analysis of the survival rates of seedlings after 14 days of *H. parallela* feeding. The different uppercase letters indicate significant differences at *P* < 0.01, as determined by Duncan’s multiple-range test

After 4, 6 and 8 days of third-instar *H. parallela* larval feeding, extensive damage was incurred on the non-transgenic control plants (Fig. 5a). The eight independent *cry8*-like transgenic lines showed a high degree of plant survival after 8 days of feeding, and the maximum number of larvae were paralyzed after 4 days of feeding on these lines. The fresh weight of the transgenic lines was also greater than that of the controls, with the weight of the Jinong 28 controls being significantly lower (78%) than that of the eight transgenic lines (Fig. 5b). We also investigated the effects of *H. parallela* feeding stress on the growth of the transgenic plants. After 2 days of *H. parallela* larval feeding on V_2_-stage plants, the leaves of the non-transgenic controls started to wilt, whereas the *cry8*-like transgenic soybean lines continued to grow well. After 4 days of feeding, the leaves of the controls were dry, yellow, and dead, whereas most leaves of the transgenic soybean plants remained green for 1 week. After 2 weeks of third-instar *H. parallela* feeding, all non-transgenic control plants had been eaten by the larvae, and the eight *cry8*-like transgenic lines displayed enhanced tolerance to *H. parallela* (Fig. 5c). We detected higher seedling survival rates on the transgenic plants than on the non-transgenic controls subjected to larval feeding. The survival rate of the non-transgenic controls was 92% lower than the survival rate of the eight transgenic lines (Fig. 5d). These results further demonstrated that, compared with the controls the *cry8*-like transgenic lines displayed increased tolerance to *H. parallela*.

### Insect bioassays

**Fig. 6:**
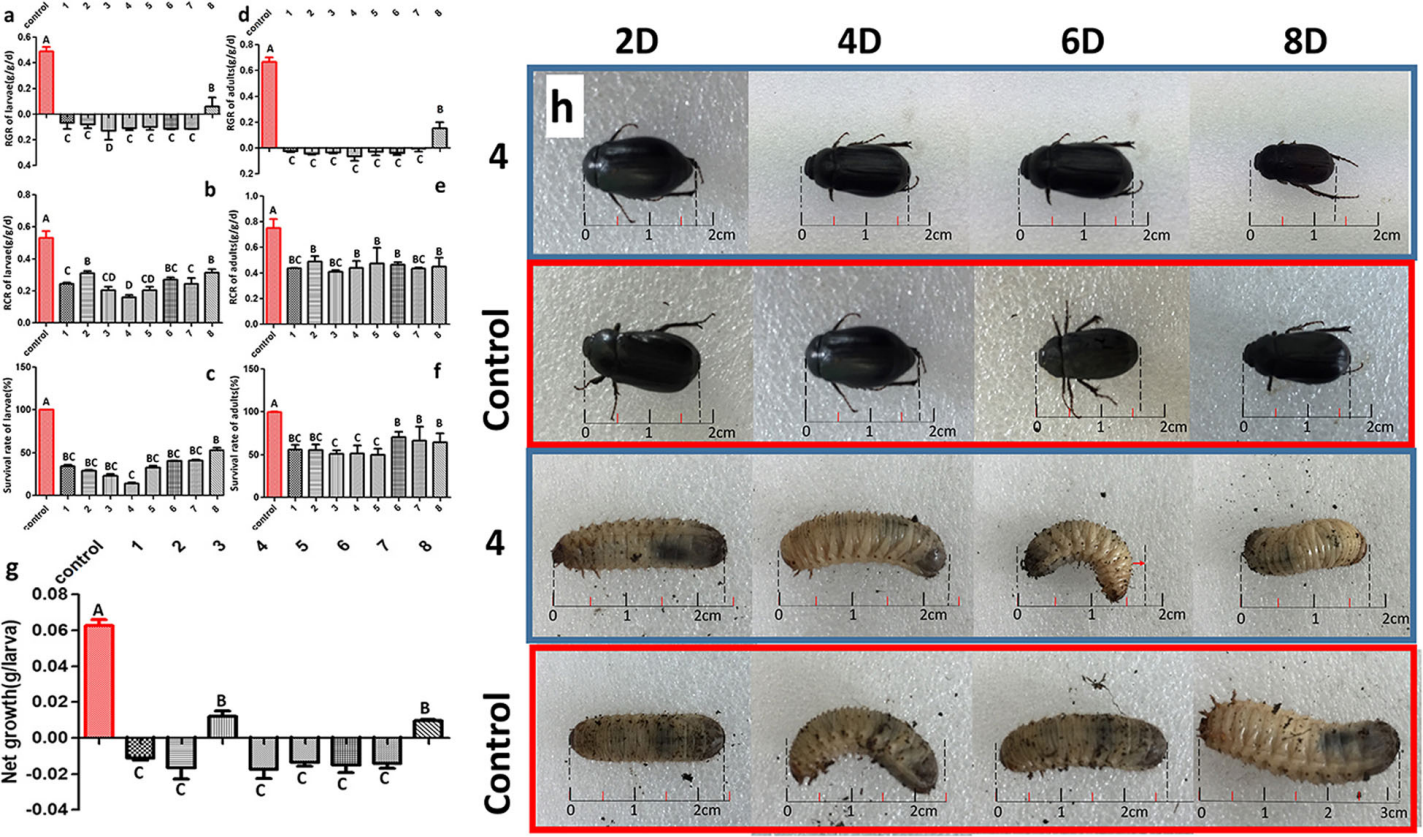
Insect bioassays. RGR(**a**), RCR (**b**) and survival rate (**c**) of *H. parallela* larvae consuming the transgenic lines after 8 days. The RGR (**d**), RCR (**e**), and survival rate (**f**) of *H. parallela* adults were also investigated. The red zone represents the Jinong 28 controls. 1–8 indicate the eight independent *cry8*-like transgenic soybean plants. (**g**) Net growth of larvae after 8 days of feeding. (**h**) The size of *H. parallela* larvae and adults after 8 days. 4: The size of *H. parallela* consuming Jinong 28-cry-4 transgenic lines; The different uppercase letters indicate significant differences at *P* < 0.01

After *H. parallela* fed on leaves or roots of the eight transgenic plants for 8 days, the relative growth rate (RGR), relative consumption rate (RCR), and survival rate were determined. The RGR (Fig. 6a) and the RCR (Fig. 6b) of the larvae feeding on the roots of the eight independent *cry8-*like transgenic lines were significantly lower than those feeding on the roots of the non-transgenic controls. The survival rates of the larvae consuming the roots of the transgenic lines declined, whereas the survival rates of the larvae consuming the roots of the non-transgenic controls were significantly higher (100% survival) (Fig. 6c). The RGR, RCR and survival rate of *H. parallela* adults feeding on the leaves were also investigated, the RGR and RCR values of the *H. parallela* adults from the *cry8-*like transgenic soybean treatments were significantly lower than those of *H. parallela* adults consuming non-transgenic leaves (Fig. 6d and e, respectively). A significant difference in the survival rate of *H. parallela* adults between the *cry8-*like transgenic lines and the non-transgenic Jinong 28 controls was found (Fig. 6f). After *H. parallela* larvae fed on the roots of the transgenic lines for 8 days, the net growth rate of the larvae decreased from 0.017 to − 0.018 g/larvae (Fig. 6g), and the size of larvae decreased. However, compared with the larvae consuming the transgenic plants, the larvae consuming the non-transgenic controls had significantly higher net growth (0.06 g/larvae) (Fig. 6g), and their size also increased (Fig. 6h). Compared with that reared on the non-transgenic controls, the size of adult *H. parallela* insects reared on the transgenic plants expressing the *cry8*-like gene decreased (Fig. 6 h).

### Agronomic performance of cry8-like transgenic lines in the field

The agronomic characteristics such as pods per plant, seeds per plant, hundred-seed weight (HSW), seed weight per plant (SWPP), plant height and plot yield of the T_2_ *cry8*-like transgenic soybean plants grown in the field were compared with those of the non-transgenic plants grown in the field. The seeds of both transgenic and control plants presented 100% germination under normal growth conditions. The growth and fertility of the transgenic plants were compared with those of the control plants. All eight independent transgenic plants showed an exhibited phenotype and were fertile (Table 3). One-way ANOVA was used to test for significant differences among the different lines. Significant differences (*P* < 0.01) were detected in the growth and fertility status of the transgenic plants compared with the control plants. The average height of the eight transgenic lines under *H. parallela* stress was twice that of the non-transgenic Jinong 28 controls. The yield of the eight transgenic lines was significantly higher (4 times) than that of the non-transgenic controls, and the seed number per plant was four times that of the Jinong 28 controls.

**Table 3:** Yield of *cry8*-like transgenic soybean plants in the field

Line	Pods per plant	Seed number per plant	hundred-seed weight (g)	seed weight per plant (g)	Average plant height (cm)	Plot yield (g)
Jinong28	30A	100A	10.91A	16.4A	40.292A	324.84A
Jinong28-cry-1	78B	222B	19.71B	35.4B	106.021B	1319.80B
Jinong28-cry-2	70B	204B	19.43B	34.9B	106.893B	1307.28B
Jinong28-cry-3	72B	188B	19.82B	34.0B	102.397B	1364.52B
Jinong28-cry-4	70B	235B	19.29B	38.2B	105.899B	1342.20B
Jinong28-cry-5	71B	215B	19.459B	32.5B	101.180B	1314.4B
Jinong28-cry-6	69B	210B	28.79B	32.5B	104.010B	1322.24B
Jinong28-cry-7	70B	218B	20.51B	32.2B	100.887B	1375.24B
Jinong28-cry-8	68B	200B	21.43B	32.6B	104.897B	1326.24B
F values	80.534	8.483	6.413	5.312	250.451	253.187
*P* values	0.000	0.002	0.006	0.005	0.000	0.000

Note: Different upper letters indicate significant differences at (*P* < 0.01)

## Discussion

Insect pests are a major limitation to soybean production [12]. *H. parallela* is a beetle belonging to the Scarabaeoidea (subfamily Melolonthidae) and causes considerable damage in soybean production worldwide [13]. *H. parallela* larvae are difficult to control with chemical insecticides because the larvae reside in the soil.

Cry toxins (such as *cry1F*, *cry2A* and *cry8*) have been widely used in genetically modified organisms for pest control because the toxic effects are based on the disruption of midgut cells within insect pests [14, 15]. Transgenic soybeans that carry multiple *Bt* genes that provide resistance to important insect pests in agricultural crops have been produced [16, 17]. Numerous studies have indicated that Bt proteins can suppress major pests thus reducing insecticide use [18, 19]. In 1994, a paper described the engineering of *cry* gene into soybean plants and compared the insecticidal activity of soybean plants [20]. In 2000, A transgenic lineage of the soybean ‘Jack’ expressing a synthetic *cry1Ac* gene (Jack-Bt) was evaluated for resistance to lepidopteran pests, compared with untransformed soybean, Jack-Bt showed three to five times less defoliation from corn earworm [21]. In 2013, a study showed that the *Cry1Ac-*expressing *Bt* soybean provided good protection against *Helicoverpa armigera*, while, limited resistance was also found for transgenic soybean against pest [22]. Most studies of *Bt* transgenic soybean have shown promising insecticidal activity against *lepidopteran* pests, not against *Scarabaeidae* pest [22]. The *Bt* strain HBF-18 (CGMCC2070), which carries two novel *cry* genes (*cry8*-like and *cry8Ga*) [23], has previously been reported to exhibit toxicity to *H. parallela* [24]. Compared with *cry8Ga*, *Cry8*-like exhibits 3-fold greater toxicity [23], confirming that *cry8*-like would be the best choice for expression in transgenic soybeans. These findings suggest that the insertion of an exogenous *cry8*-like gene into soybean is a feasible approach to produce insect resistance.

In this study, we successfully inserted the *cry8*-like gene into soybean to confer resistance against *H. parallela* to transgenic plants. We obtained eight independent transgenic soybean lines, each containing a single copy of the *cry8-*like gene. In the case of soybean, leaves and roots are the major plant parts damaged by *H. parallela*. Therefore, a high level of expression of Bt protein in soybean leaves and roots is important to obtain effective pest control. The present ELISA results showed that the Cry8-like levels in the two *Bt* soybean lines were relatively high, ranging from 16 to 19 ng·mg^− 1^. Although levels did not reach those previously reported [25], by comparison with untransformed Jinong28, the transgenic lines showed five times greater resistance than Jinong28 to artificially infestations of *H. parallela*. In conventional field plots at one location in 2018, transgenic soybean plants also showed four times greater resistance than untransformed Jinong28 to natural infestations of *H. parallela*. According to Walker et al. [21], a transgenic line Jack-Bt also showed four times greater resistance than untransformed Jack to natural infestations of lesser cornstalk borer. The results of the bioassay revealed a significant difference in the survival of *H. parallela* reared on the eight independent transgenic soybean lines. The survival rate of *H. parallela* consuming the Jinong 28-cry-4 transgenic line was the lowest (14%), while, the survival rate of *H. parallela* consuming the Jinong 28-cry-8 transgenic line was the highest (57%). Accordingly, the Jinong 28-cry-4 transgenic line had the highest accumulation of Bt protein, and the Jinong 28-cry-8 transgenic line had the lowest accumulation of Bt protein. These results suggest that the efficacy of insect protection was based on the level of protein accumulation in the transgenic plants. The variable insect mortality levels was mostly in agreement with the protein expression levels in the transgenic plants, and the protein expression levels in the transgenic plants was related to the intensity of expression of the *cry8-*like gene. The variation in transgene expression has been attributed to several factors, including differences with respect to chromosome location, copy number, and transgene construct fidelity. We investigated the transcriptional expression of the *cry8*-like gene in the eight independent transgenic soybean lines. All transgenic plants showed variable levels of *cry8*-like expression. The *cry8*-like construct was probably inserted at different locations in the plant genome, thus producing differences in Cry toxin concentration, as previously suggested in [22].

Both the cumulative and relative food consumption rates of the *H. parallela* larvae and adults feeding on the transgenic soybean plants were significantly reduced compared to those of the larvae feeding on the control plants. The mortality of *H. parallela* larvae mostly reached 100% after 2 weeks of feeding, and that of adults was 85%. The results showed that the transgenic plants exerted differential effects on the larvae and adults and that the larvae seemed to be more sensitive to the Bt toxin than the adults. Some researchers also reported that the first and second instar are the most sensitive to Cry protein [26]. The high toxicity of *Cry8-*like to *H. parallela* larvae can be related to the natural tolerance of insects to the Bt proteins.

The evolution of insect resistance directly threatens Bt toxins efficacy in the transgenic plants [27]. The transgenic soybean plants expressing a *Cry1Ac* gene that showed a poor control of *Spodoptera* species is one example [28], as well as the western corn rootworm showing resistance to *Cry3Bb1* maize in the USA [29]. Therefore, the transgenic crops including two or more Bt toxins, and novel Bt toxins are considered better options [30]. In our experiment, the novel *cry8-*type gene was used in the transgenic soybeans for the control of *Scarabaeidae* beetles. In general, our results showed that transgenic soybean plants expressing the *cry8*-like gene are resistant to *H. parallela*, but a limitation of the study is that only one *cry* gene was used. Therefore, as already suggested in [22], future research should focus on the multiple-gene Bt transgenic soybeans to delay the fast development of resistance in the pest.

## Conclusions

In summary, our study demonstrated that *cry8*-like expression is an effective technique for improving resistance to *H. parallela* in soybean. The eight lines developed in this study showed enhanced resistance to *H. parallela* adults and larvae. In addition, these lines did not have any negative impact on the agronomic traits studied.

## Methods

### Biological materials

The 1971-bp cDNA of the *cry8-*like gene from the *Bacillus thuringiensis* strain HBF-18 (from the Jilin Academy of Agricultural Sciences) was ligated into the *Bam*HI*-Sac*I site of pCAMBIA3300 [31] to place the coding region under the regulatory control of the 35S promoter and the *Nos* terminator. The recombinant plasmid was named pCAMBIA3300-cry8-like and introduced into *Agrobacterium tumefaciens* strain LBA4404 via the freeze-thaw method [32]. We introduced *cry8*-like from the *Bt* strain HBF-18 (from the Jilin Academy of Agricultural Sciences) into the soybean cultivar Jinong 28 (Approval number 2011010), the seeds of Jinong 28 were provided by the Biotechnology Center of Jilin Agricultural University. Jinong 28 has good agronomic characteristics but no resistance to *H. parallela.* We used *Agrobacterium*-mediated transformation [33] to transform callus of the soybean cultivar Jinong 28. The transformation process was divided into five sequential steps: bacterial inoculation, cocultivation, resting, selection and plant regeneration. Eight independent transgenic lines were harvested and propagated in the greenhouse: Jinong 28-cry-1, Jinong 28-cry-2, Jinong 28-cry-3, Jinong 28-cry-4, Jinong 28-cry-5, Jinong 28-cry-6, Jinong 28-cry-7, and Jinong 28-cry-8. These transformed plants were used as sources of plant materials. Untransformed plants of cultivar Jinong 28 were used as controls. T_0_ seeds from the primary transformants were harvested and analyzed for the identification of transformants.

### Polymerase chain reaction analysis

Genomic DNA from T_0_ and T_1_ plants and from the controls was extracted from the leaf tissues following the method of Kim and Hamada [34]. Quantification of DNA was performed using a Nanodrop spectrophotometer (Thermo Fisher Scientific, Waltham, Masachusetts, USA). The genomic DNA from the T_0_ and T_1_ transgenic plants was used to perform PCR to detect the presence of the *cry8-*like gene. The specific primers for PCR were designed based on the highly conserved sequence of the *cry8-*like gene via Primer 5.0 software (Additional file 4: Table S3). The PCR conditions were as follows: 94 °C for 5 min; 40 cycles of 95 °C for 30 s, 58 °C for 30 s, and 72 °C for 30 s; and a final extension of 72 °C for 10 min. Genomic DNA from Jinong 28 was used as a negative control, and the vector was used as a positive control. The PCR products were subsequently analyzed by agarose gel electrophoresis.

### Quantitative reverse transcription-PCR

qRT-PCR analysis was performed to determine the transcript abundance of c*ry8*-like. The gene-specific primer pairs P3 (5′-TTTGGATCCAAGCTTTCTAGACCCGGGCCTAT-3′) and P4 (5′-TTTGAGCTCTCAAAGTTCATCCTTCTCGGAGT-3′) were used to amplify *cry8*-like. A *lectin* gene (GenBank: A5547–127) was used as a reference gene, which was amplified with the primer pairs P5 (5′-GCACTTAAGATACTCTAGGTAC-3′) and P6 (5′-CCACCTCCCTACTATCCATT-3′). At the three-leaf stage (V_3_), 0.3 g of leaves, 0.3 g of stems and 0.3 g of roots from each plant were washed thoroughly with ddH_2_O, and then immediately frozen in liquid nitrogen for qRT-PCR analysis. Total RNA was extracted using an RNA isolation kit (Omega Bio-tek, Norcross, GA, USA). cDNA synthesis was then performed using a reverse transcription kit (Omega Bio-tek, Norcross, GA, USA). Three biological replicates with three technical replicates of each qRT-PCR were performed. The qRT-PCR analysis was performed using a Bio-Rad CFX system (Amersham Biosciences, Little Chalfont, Buckinghamshire, UK).

### Southern blot analysis

Genomic DNA was isolated from leaf tissues as described previously. Approximately 2 μg of DNA was digested with *Bam*HI, separated on 0.8% agarose gel and then blotted to Hybond™-N+ nylon membrane (Amersham Biosciences, Little Chalfont, Buckinghamshire, UK). Purified *cry8*-like gene was used as a probe, and a DIG DNA Labeling Detection Kit (Roche Company, Basel, Swiss) was used for Southern blots according to the manufacturer’s instructions.

### Enzyme-linked immunosorbent assay (ELISA) for the quantitative estimation of the Bt protein

We analyzed 27 samples (three replicates of each of the 8 transgenic plants plus a control plant) for the presence of the Cry8-like protein with an enzyme-linked immunosorbent assay (ELISA). Non-transgenic soybean cultivar Jinong 28 was used as a control. We essentially followed a previously published protocol [11]. The BCA protein assay kit (CWBIO, Beijing, China) was used to estimate the total soluble protein from different tissues, the anti-Cry8-like antibody was prepared in a rabbit. The protein concentration was calculated according to a standard method [35]. The optical density (OD) values was measured at 450 nm wavelength using Micro-plate reader (Biocompare, South San Francisco, USA).

### Insect collecting and feeding

The *H. parallela* insects were collected from Jilin Agricultural University, Changchun, China, located at 43°48′ N, 125°24′ E. The *H. parallela* larvae were collected from soybean fields in late May and reared in a glasshouse on fresh roots of non-transgenic soybean plants. The *H. parallela* beetles were maintained in a ventilated incubator with a layer of soil (18–20% moisture) and fresh leaves as a food source. Healthy individuals were selected and starved for 24 h before use in bioassays.

### Insect bioassays

For the bioassays, *H. parallela* adults were fed leaves from the T_1_ transgenic plants, which had been confirmed to be transgenic by PCR, and the larvae were fed roots. One hundred insects were released per Petri dish containing 30 g leaves from one independent line. Each experimental unit consisted of three Petri dishes, which represent the three replicates used for each soybean line. The plates were incubated in an acclimatized room (24–26 °C, 18-h light/6-h dark cycle). The light wavelength ranged from 480 to 780 nm. The leaves and roots were replaced with fresh material every 3 days. Observations of the behavior, mortality, and other parameters were recorded daily for 15 days. The larval survival rate was calculated as N*n*/N_*0*_× 100, where N*n* is the number of larvae on day *n* of the experiment and N_*0*_ is the number of *H. parallela* larvae at the beginning of the experiment. The relative consumption rate (g/g/d) = weight of food eaten/(duration of feeding (d) × mean weight of the larvae). The weight of food consumed (g) = weight of diet after feeding (g)- weight of diet before feeding (g), and the relative growth rate (g/g/d) = weight gain of the larvae/(duration of feeding (d) × mean weight of the larvae).

### Field trial evaluations

The *cry8-*like transgenic soybean plants and Jinong 28 control plants were grown at Jilin Agricultural University, Changchun, China, located at 43°48′ N, 123°24′ E. A randomized complete block design was used. The field was divided into three blocks of 300 m^2^ (15 × 20 m), each of which was subdivided into nine subsections. Seeds were planted in nine random subsections of each block. The plants were grown under natural temperature, light and humidity conditions during the season. The climate of the region is considered semiarid, and the region is considered to be a representative region for typical soil in Northeast China. Two months after sowing, the *H. parallela* adults and third-instar larvae were released into the field (100/300 m^2^). Pesticides were not applied prophylactically at the seeding stage. The following characteristics were measured: plant height, pods per plant, seed number per plant, grain yield and hundred-seed weight. The pods of each plant were counted for 100 plants per line, and the average number was calculated. The hundred-seed weight was determined from 100 randomly chosen soybean samples, and the yield was obtained by weighing the grain mass.

### Data analysis

All data were analyzed via SPSS version 22.0 software (SPSS Inc*.*, Chicago, IL, USA) [36], each with three replicates. Two-way ANOVA was used to test whether the soybean lines or organs had a significant effect on the accumulation of the Cry8-like protein and the *cry8*-like gene in the transgenic lines. These analyses of variance were performed in two steps. Two-way ANOVAs (including all interactions and simple and main effects) were used to test for significant differences among the transgenic lines. The means were subsequently tested a posteriori to the ANOVAs via Duncan’s test at the 0.05 probability level. One-way ANOVA in conjunction with Student’s *t* test was used to determine whether the differences in the agronomic characteristics between the eight different transgenic lines were significant. *P <* 0.01 was considered statistically significant.

## Supplementary information

**Additional file 1: Fig. S1.** Detection of the different *cry8*-like transgene regions in the eight transgenic lines by PCR.

**Additional file 2: Table S1.** *S*ignificant differences among transgenic soybean lines.

**Additional file 3: Table S2.** *S*ignificant differences among plant organs.

**Additional file 4: Table S3.** Primers used for the experiments in this study.

## Data Availability

All the data or materials used during this study are available from the corresponding author upon reasonable request.

## Abbreviations

d: Day
ELISA: Enzyme-linked immunosorbent assay
H: Hour
HSW: Hundred-seed weight
qRT-PCR: Quantitative reverse transcription-polymerase chain reaction
RCR: Relative consumption rate
RGR: Relative growth rate
SWPP: Seed weight per plant

## References

[1] Hill AM, Katcher HI, Flickinger BD, Kris-Etherton PM (2008). Human nutrition value of soybean oil and soy protein.

[2] Wang C-L (1980). Soybean insects occurring at podding stage in Taichung. J Agric Res China.

[3] Shi S, Cui J, Song P, Zang D, Li W, Wu T (2014). Occurring regularity of *Holotrichia parallela* in summer soybean field. Soybean Sci.

[4] Bravo A, Gómez I, Porta H, García-Gómez BI, Rodriguez-Almazan C, Pardo L (2013). Evolution of *Bacillus thuringiensis* cry toxins insecticidal activity. Microb Biotechnol.

[5] Kumar S, Chandra A, Pandey KC (2008). *Bacillus thuringiensis* (Bt) transgenic crop: an environment friendly insect-pest management strategy. J Environ Biol.

[6] Ostlie K, Hutchison WD, Hellmich R (1997). Bt corn and European corn borer.

[7] Xue J, Liang GM, Crickmore N, Li H, He K, Song FP (2008). Cloning and characterization of a novel cry1A toxin from *Bacillus thuringiensis* with high toxicity to the Asian corn borer and other lepidopteran insects. FEMS Microbiol Lett.

[8] Burlet A, Chapleur-Chateau M, Haumont-Pellegri B, Jansen F, Menzaghi F, Fernette B (1998). Characterization of cry genes in a Mexican *Bacillus thuringiensis* strain collection. Appl Environ Microbiol.

[9] Anilkumar KJ, Rodrigo-Simon A, Ferre J, Pusztai-Carey M, Sivasupramaniam S, Moar WJ (2008). Production and characterization of *Bacillus thuringiensis* cry1Ac-resistant cotton bollworm *Helicoverpa zea* (Boddie). Appl Environ Microbiol.

[10] Shu C, Yan G, Wang R, Zhang J, Feng S, Huang D (2009). Characterization of a novel cry8 gene specific to *Melolonthidae* pests: *Holotrichia oblita* and *Holotrichia parallela*. Appl Microbiol Biotechnol.

[11] Geng L, Chi J, Shu C, Gresshoff PM, Song F, Huang D (2013). A chimeric cry8Ea1 gene flanked by MARs efficiently controls *Holotrichia parallela*. Plant Cell Rep.

[12] Rupe J, Luttrell RG, Johnson LA, White PJ, Galloway R (2008). Effect of pests and diseases on soybean quality. Soybeans: chemistry, production processing, and utilization.

[13] Kogan M, Irwin M, Sinclair J, Slife F (1997). Major world soybean diseases, weeds and insect pests: a diagnostic pictorial atlas.

[14] Bandyopadhyay D (2018). Genetically modified crops, agriculture and biosafety. Securing our natural wealth.

[15] Romeis J, Meissle M, Bigler F (2006). Transgenic crops expressing *Bacillus thuringiensis* toxins and biological control. Nat Biotechnol.

[16] Roh JY, Choi JY, Li MS, Jin BR, Je YH (2007). *Bacillus thuringiensis* as a specific, safe, and effective tool for insect pest control. J Microbiol Biotechnol.

[17] Datta K, Vasquez A, Tu J, Torrizo L, Alam MF, Oliva N (1998). Constitutive and tissue-specific differential expression of the *cryIA(b)* gene in transgenic rice plants conferring resistance to rice insect pest. Theor Appl Genet.

[18] Lu Y, Wu K, Jiang Y, Guo Y, Desneux N (2012). Widespread adoption of Bt cotton and insecticide decrease promotes biocontrol services. Nature..

[19] Bravo A, Likitvivatanavong S, Gill SS, Soberon M (2011). *Bacillus thuringiensis*: a story of a successful bioinsecticide. Insect Biochem Mol Biol.

[20] Parrott WA, All JN, Adang MJ (1994). Recovery and evaluation of soybean plants transgenic for a *Bacillus thuringiensis* var. Kurstaki insecticidal gene. In Vitro Cell Dev Biol Plant.

[21] Walker DR, All JN, McPherson RM (2000). Field evaluation of soybean engineered with a synthetic *cry1Ac* transgene for resistance to corn earworm, soybean looper, velvetbean caterpillar (Lepidoptera: Noctuidae), and lesser cornstalk borer (Lepidoptera: Pyralidae). J Econ Entomol.

[22] Yu H, Li Y, Li X (2013). Expression of *Cry1Ac* in transgenic Bt soybean lines and their efficiency in controlling lepidopteran pests. Pest Manag Sci.

[23] Jiang J, Huang Y, Shu C, Soberon M, Bravo A, Liu C (2017). *Holotrichia oblita* midgut proteins that bind to *Bacillus thuringiensis* cry8-like toxin and assembly of the *H. oblita* midgut tissue transcriptome. Appl Environ Microbiol.

[24] Bi Y, Zhang Y, Shu C, Crickmore N, Wang Q, Du L (2015). Genomic sequencing identifies novel *Bacillus thuringiensis* Vip1/Vip2 binary and cry8 toxins that have high toxicity to *Scarabaeoidea* larvae. Appl Microbiol Biotechnol.

[25] Macrae TC, Baur ME, Boethel DJ (2005). Laboratory and field evaluations of transgenic soybean exhibiting high-dose expression of a synthetic *Bacillus thuringiensis cry1A* gene for control of Lepidoptera. J Econ Entomol.

[26] Shanmugam PS, Balagurunathan R, Sathiah N (2006). Susceptibility of *Helicoverpa armigera* Hübner instars to *Bacillus thuringiensis* insecticidal crystal proteins. Pestic Res J.

[27] Soberón M, Pardo-López L, López I (2007). Engineering modified Bt toxins to counter insect resistance. Science..

[28] Bernardi O, Sorgatto RJ, Barbosa AD (2014). Low susceptibility of *Spodoptera cosmioides, Spodoptera eridania* and *Spodoptera frugiperda* (Lepidoptera: Noctuidae) to genetically-modified soybean expressing Cry1Ac protein. Crop Prot.

[29] Gassmann AJ, Petzold-Maxwell JL, Keweshan RS (2011). Field-evolved resistance to Bt maize by western corn rootworm. PLoS One.

[30] Sheikh AA, Wani MA, Bano P (2017). An overview on resistance of insect pests against Bt crops. J Entomol Zool Stud.

[31] Wu N, Wang P, Lin N, Lu S, Feng Y, Rong J (2017). Construction of a chalcone reductase expression vector and transformation of soybean plants. Mol Med Rep.

[32] Wang X-Q, Shen X, He Y-M, Ren T-N, Wu W-T, Xi T (2011). An optimized freeze-thaw method for transformation of *Agrobacterium tumefaciens* EHA 105 and LBA 4404. Pharm Biotechnol.

[33] Donaldson PA, Simmonds DH (2000). Susceptibility to *Agrobacterium tumefaciens* and cotyledonary node transformation in short-season soybean. Plant Cell Rep.

[34] Kim SH, Hamada T (2005). Rapid and reliable method of extracting DNA and RNA from sweetpotato, Ipomoea batatas (L). Lam Biotechnol Lett.

[35] Palmer HM (2000). Using antibodies: a laboratory manual. J Antimicrob Chemother.

[36] Verma JP (2013). Data analysis in management with SPSS software.

