# Supplementary material for: Soybean plants expressing the Bacillus thuringiensis cry8-like gene show resistance to Holotrichia parallela
Source: BMC Biotechnol. 2019 Oct 15;19:66. doi: 10.1186/s12896-019-0563-1 (PMC6794784; doi:10.1186/s12896-019-0563-1)
Supplement: Supplementary file 1 — Additional file 1: Fig. S1. Detection of the different cry8-like transgene regions in the eight transgenic lines by PCR. [file 12896_2019_563_MOESM1_ESM.pptx]

## Slide 1
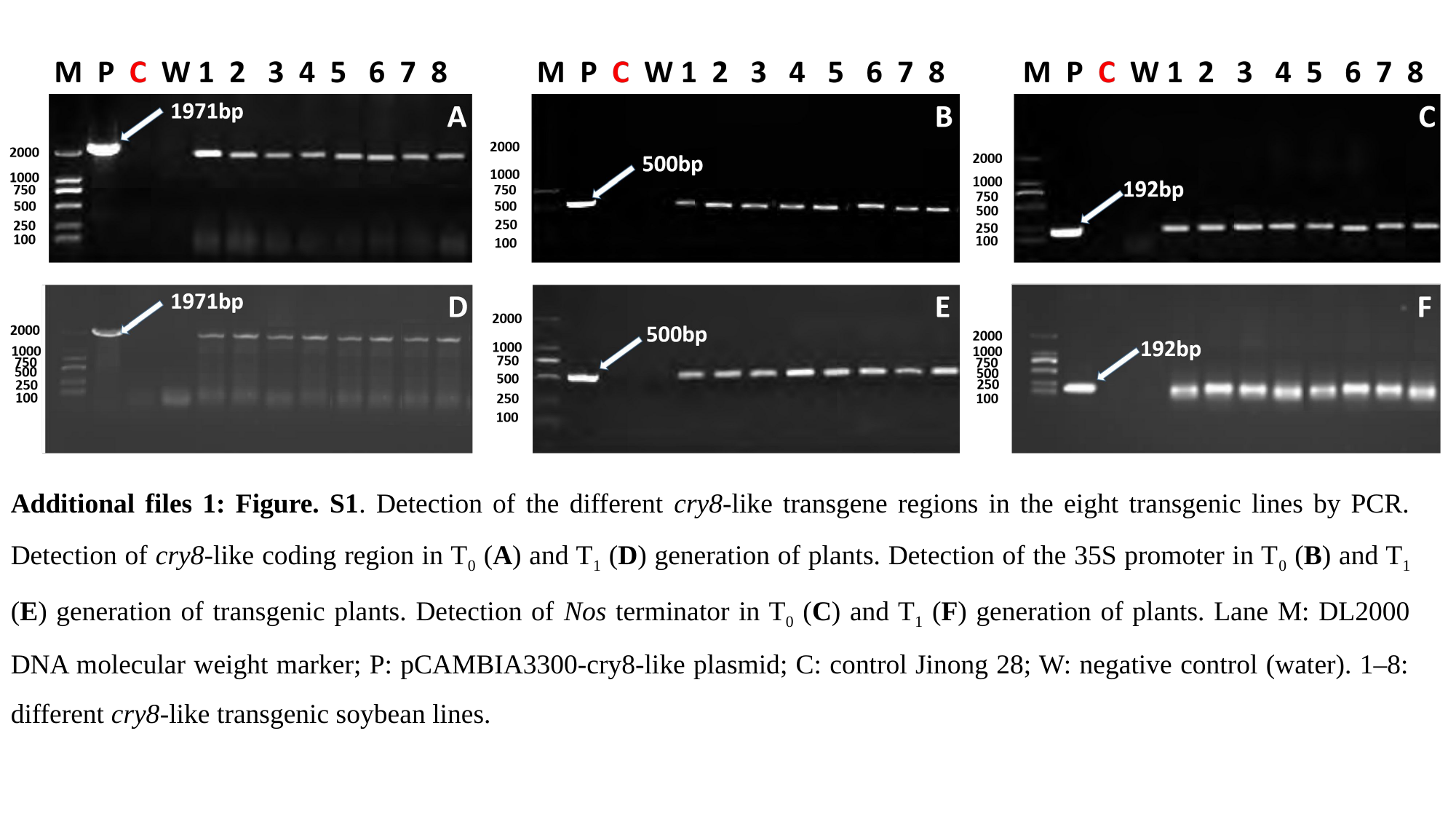

Additional files 1: Figure. S1. Detection of the different cry8-like transgene regions in the eight transgenic lines by PCR. Detection of cry8-like coding region in T0 (A) and T1 (D) generation of plants. Detection of the 35S promoter in T0 (B) and T1 (E) generation of transgenic plants. Detection of Nos terminator in T0 (C) and T1 (F) generation of plants. Lane M: DL2000 DNA molecular weight marker; P: pCAMBIA3300-cry8-like plasmid; C: control Jinong 28; W: negative control (water). 1–8: different cry8-like transgenic soybean lines.
