# Supplementary material for: Soybean plants expressing the Bacillus thuringiensis cry8-like gene show resistance to Holotrichia parallela
Source: BMC Biotechnol. 2019 Oct 15;19:66. doi: 10.1186/s12896-019-0563-1 (PMC6794784; doi:10.1186/s12896-019-0563-1)
Supplement: Supplementary file 2 — Additional file 2: Table S1. Significant differences among transgenic soybean lines. [file 12896_2019_563_MOESM2_ESM.pdf]

Table S1. Significant differences among transgenic soybean lines.

			subset				
	Lines	Number	1	2	3	4	5
Duncan[a,b]	Control	6	0.0000				
	Jinong28-cry-8	6		6.3333			
	Jinong28-cry-6	6		7.1667			
	Jinong28-cry-7	6			9.6667		
	Jinong28-cry-1	6			11.1667		
	Jinong28-cry-2	6			11.5000		
	Jinong28-cry-3	6				14.5000	
	Jinong28-cry-5	6				15.0000	15.0000
	Jinong28-cry-4	6					16.5000
	Significance		1.000	0.345	0.052	0.569	0.093

Note: Two-way ANOVAs using Duncan test at the 0.05 probability level.
