# Supplementary material for: Soybean plants expressing the Bacillus thuringiensis cry8-like gene show resistance to Holotrichia parallela
Source: BMC Biotechnol. 2019 Oct 15;19:66. doi: 10.1186/s12896-019-0563-1 (PMC6794784; doi:10.1186/s12896-019-0563-1)
Supplement: Supplementary file 3 — Additional file 3: Table S2. Significant differences among plant organs. [file 12896_2019_563_MOESM3_ESM.pdf]

Table S2. Significant differences among plant organs.

			subset		
	Organs	Number	1	2	3
Duncan[a,b]	stems	18	8.8333		
	roots	18		10.2222	
	leaves	18			11.5556
	Significance		1.000	1.000	1.000

Note: Two-way ANOVAs using Duncan test at the 0.05 probability level.
