# Supplementary material for: Soybean plants expressing the Bacillus thuringiensis cry8-like gene show resistance to Holotrichia parallela
Source: BMC Biotechnol. 2019 Oct 15;19:66. doi: 10.1186/s12896-019-0563-1 (PMC6794784; doi:10.1186/s12896-019-0563-1)
Supplement: Supplementary file 4 — Additional file 4: Table S3. Primers used for the experiments in this study. [file 12896_2019_563_MOESM4_ESM.pdf]

Table S3. Primers used for the experiments in this study.

Primer name	Sequences of Primer (5' to 3')	Sequences of reverse Primer (5' to 3')
**Cry**	**TTTGGATCCAAGCTTTCTAGACCCGGGCCTAT**	**TTTGAGCTCTCAAAGTTCATCCTTCTCGGAGT**
**35S**	**TAGAGGACCTAACAGAAC**	**CCGTGTTCTCTCCAAATG**
**Nos**	**GAATCCTGTTGCCGGTCTTG**	**TTATCCTAGTTTGCGCGCTA**

Note: **35S** is promoter, ***Nos*** is Terminator.
